# Supplementary material for: Systemic Ablation of Camkk2 Impairs Metastatic Colonization and Improves Insulin Sensitivity in TRAMP Mice: Evidence for Cancer Cell-Extrinsic CAMKK2 Functions in Prostate Cancer
Source: Cells. 2022 Jun 10;11(12):1890. doi: 10.3390/cells11121890 (PMC9221545; doi:10.3390/cells11121890)
Supplement: Supplementary file 1 [file cells-11-01890-s001.zip › Supplementary File S1.pdf]

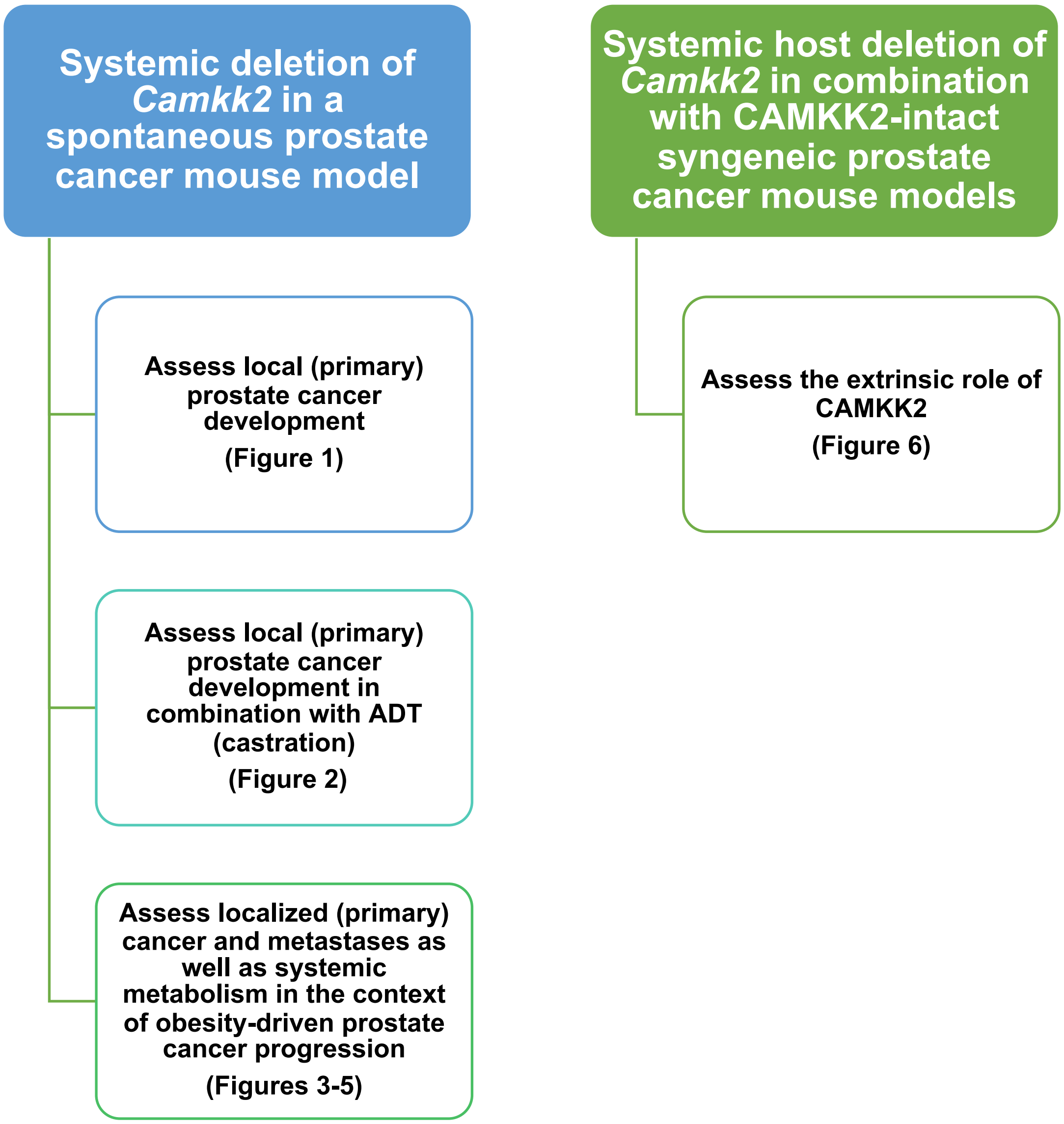

Supplemental Figure S1: Workflow of study.

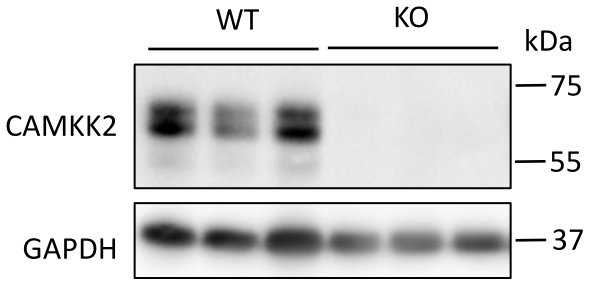

Supplemental Figure S2: Germline *Camkk2* knockout in C57BL/6 mice confirmed by western blot analysis of brain lysates from *Camkk2*$^{+/+}$ (WT) and *Camkk2*$^{-/-}$ (KO) mice.

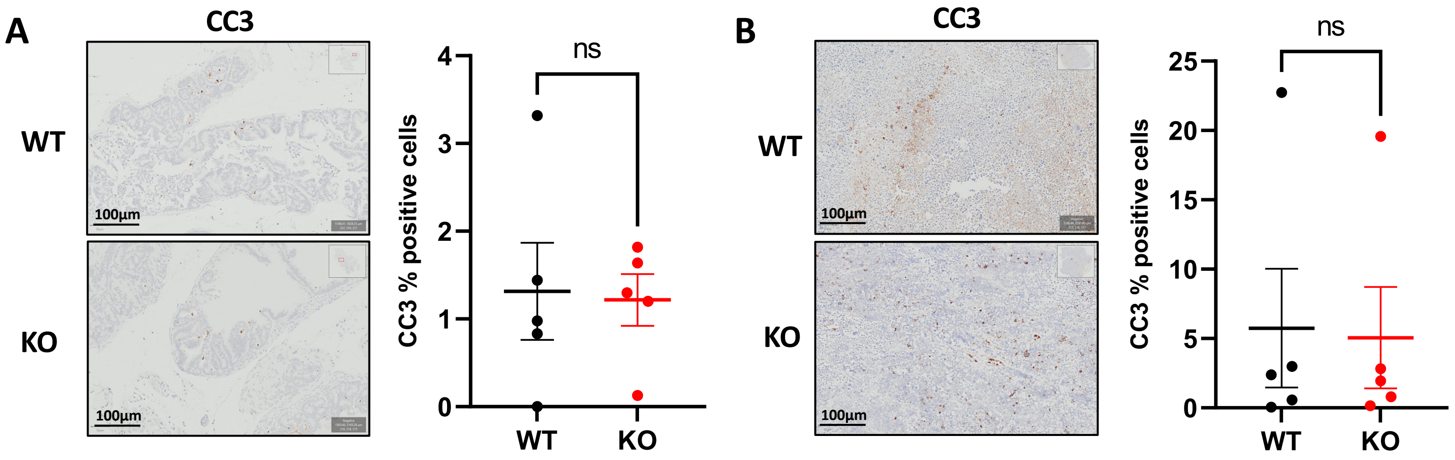

Supplemental Figure S3: *Camkk2* knockout did not affect apoptosis as measured by cleaved caspase-3 (CC3) staining in prostates/tumors from TRAMP mice. Cleaved caspase-3 (CC3) staining of prostate/tumors at (A) 15 weeks (WT *n*=5, KO *n*=5) and (B) 30 weeks (WT *n*=5, KO *n*=5) shows no significant change in staining. ns = not significant by *t* test.

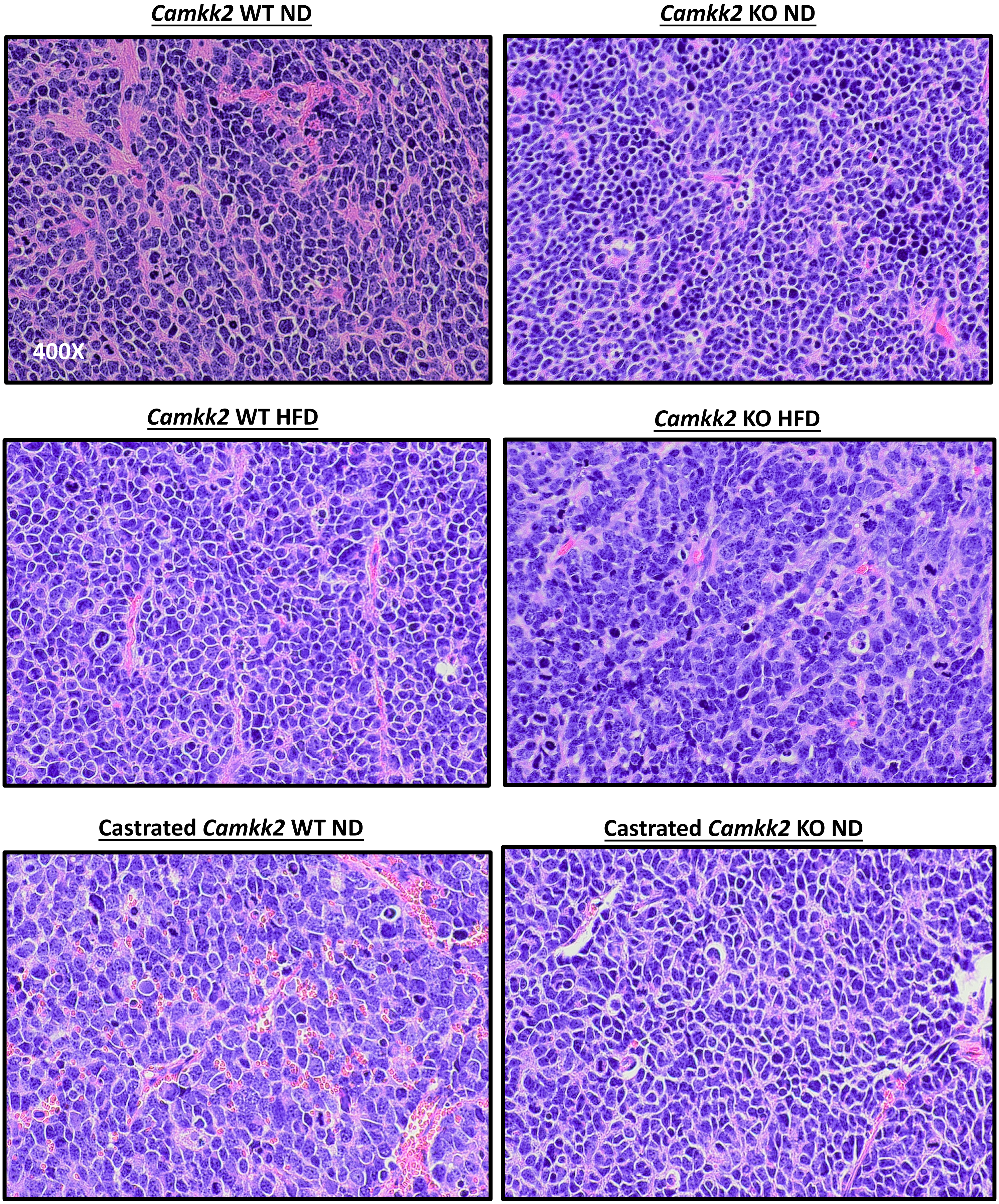

Supplemental Figure S4: Tumors exhibit classic NEPC morphology at 30 weeks. Representative high-magnification images are shown of tumors from multiple 30-week old TRAMP mouse cohorts used throughout the study demonstrating characteristic features of NEPC such as reduced cytoplasm, nuclear molding, and granular chromatin. NEPC phenotypes were confirmed by a pathologist (M. Ittmann).

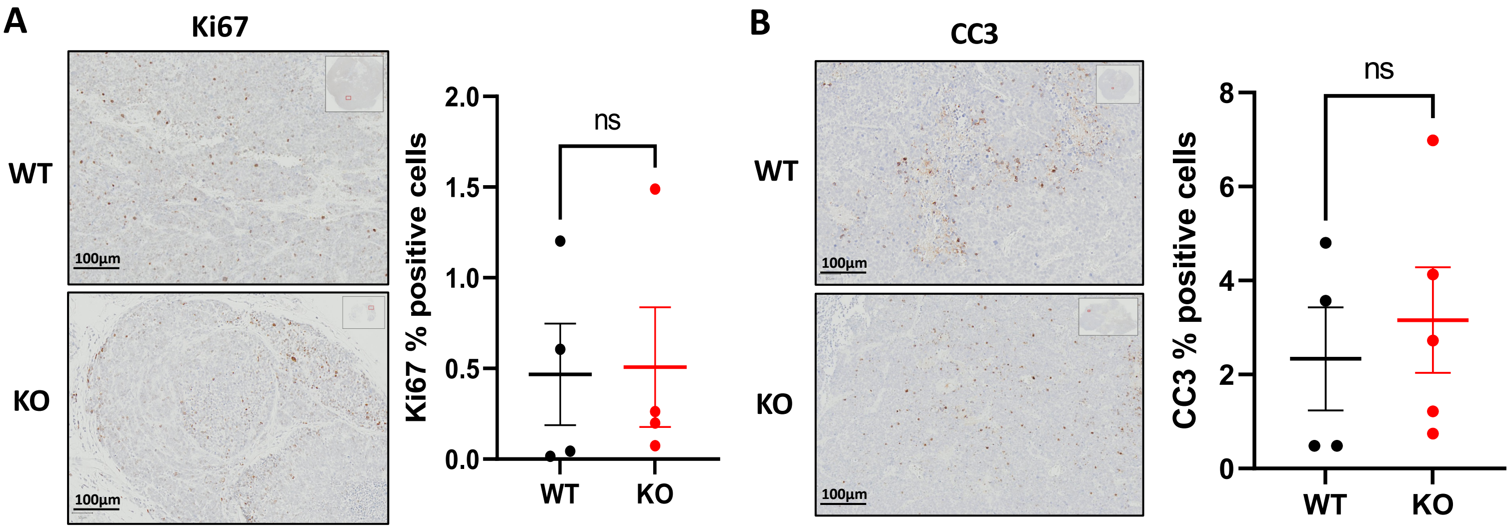

Supplemental Figure S5: Effects of *Camkk2* knockout in castrated TRAMP mice on proliferation and apoptosis. (A) Ki67 (WT *n*=4, KO *n*=4) and (B) cleaved caspase-3 (CC3) IHC analyses of castrated TRAMP mice (WT *n*=4, KO *n*=5). ns = not significant by *t* test.

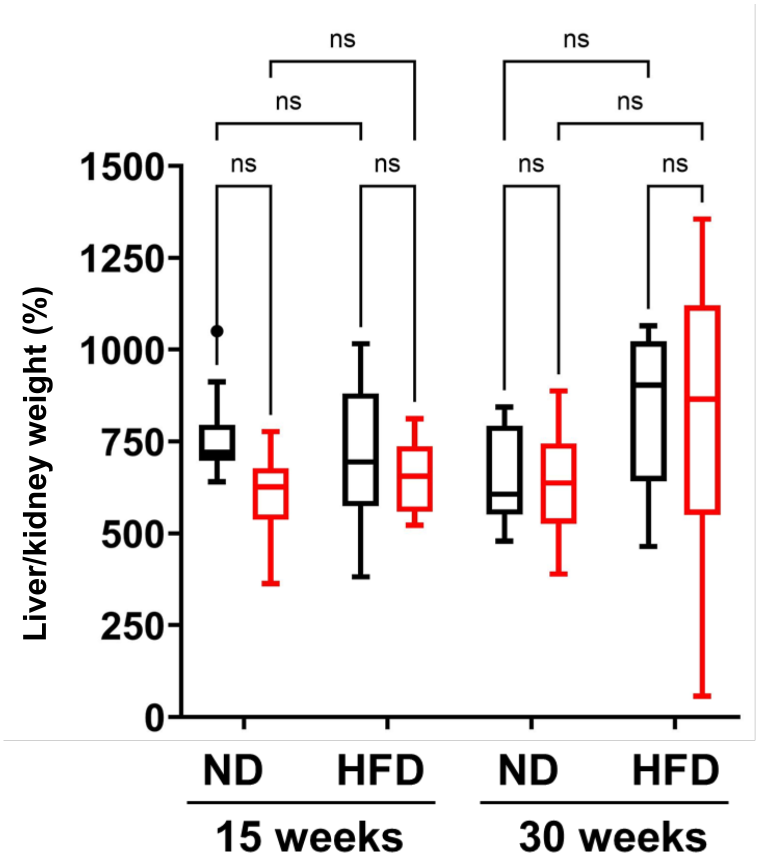

Supplemental Figure S6: CAMKK2 status did not influence liver weights in TRAMP mice. Livers were weighed and normalized to kidney weights in 15- and 30-week old TRAMP;*Camkk2*$^{+/+}$ (black) and TRAMP;*Camkk2*$^{-/-}$ (red) mice fed normal (ND) or high-fat diet (HFD) (15 weeks ND: WT *n*=10, KO *n*=10; 15 weeks HFD: WT *n*=10, KO *n*=8; 30 weeks ND: WT *n*=10, KO *n*=9; 30 weeks HFD: WT *n*=10, KO *n*=9). ns = no significance; Two-way ANOVA.

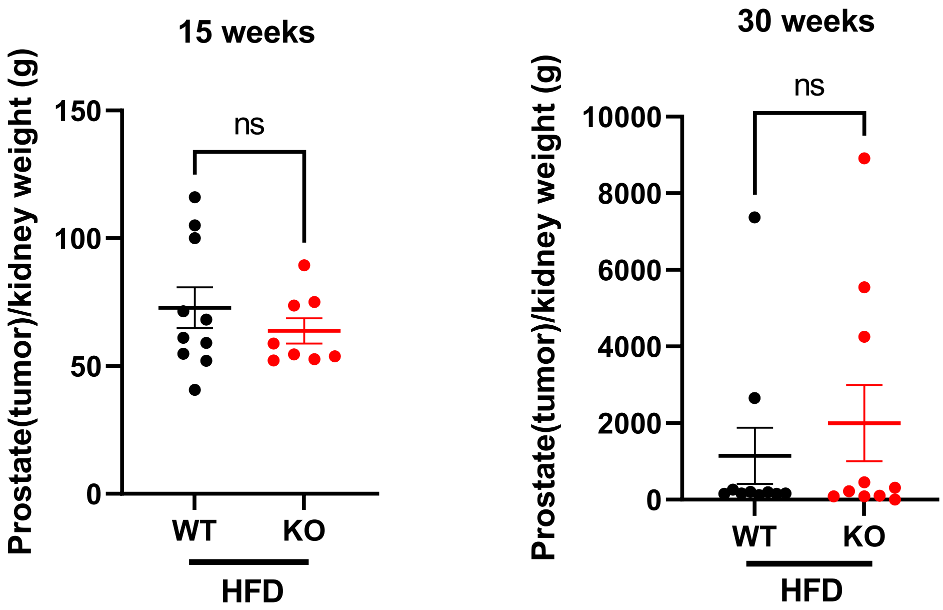

Supplemental Figure S7: *Camkk2* knockout does not impact prostate/primary tumor weights in high-fat diet (HFD)-fed mice. Prostate/primary tumor weights normalized to kidney weight at 15 (left) or 30 (right) weeks in HFD-fed mice (15-week HFD WT *n*=10; 15-week HFD KO *n*=8; 30-week HFD WT *n*=11; 30-week HFD KO *n*=10). ns = no significance.

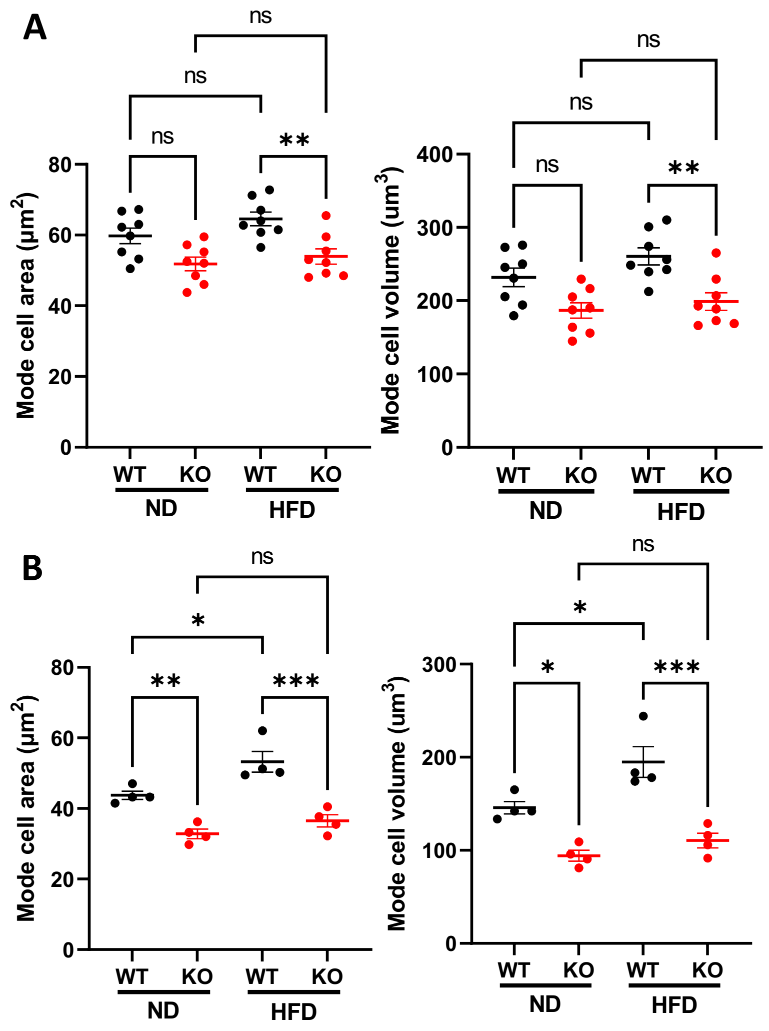

Supplemental Figure S8: Host *Camkk2* ablation decreased cancer cell size in TRAMP mice. (A) Mode of individual cell areas and volumes for tumors derived from representative TRAMP;*Camkk2*$^{+/+}$ and TRAMP;*Camkk2*$^{-/-}$ mice at 30 weeks (*n*=5/group). (B) Mode of individual cell areas and volumes for lung metastases derived from representative TRAMP;*Camkk2*$^{+/+}$ and TRAMP;*Camkk2*$^{-/-}$ mice at 30 weeks (*n*=3/group). ns=not significant; **P*<0.05 ; ***P*<0.01; ****P*<0.001. For morphometric analysis, *n*>1 x 10^6 cells/data point. Average cell areas and volumes are shown in Figure 5.

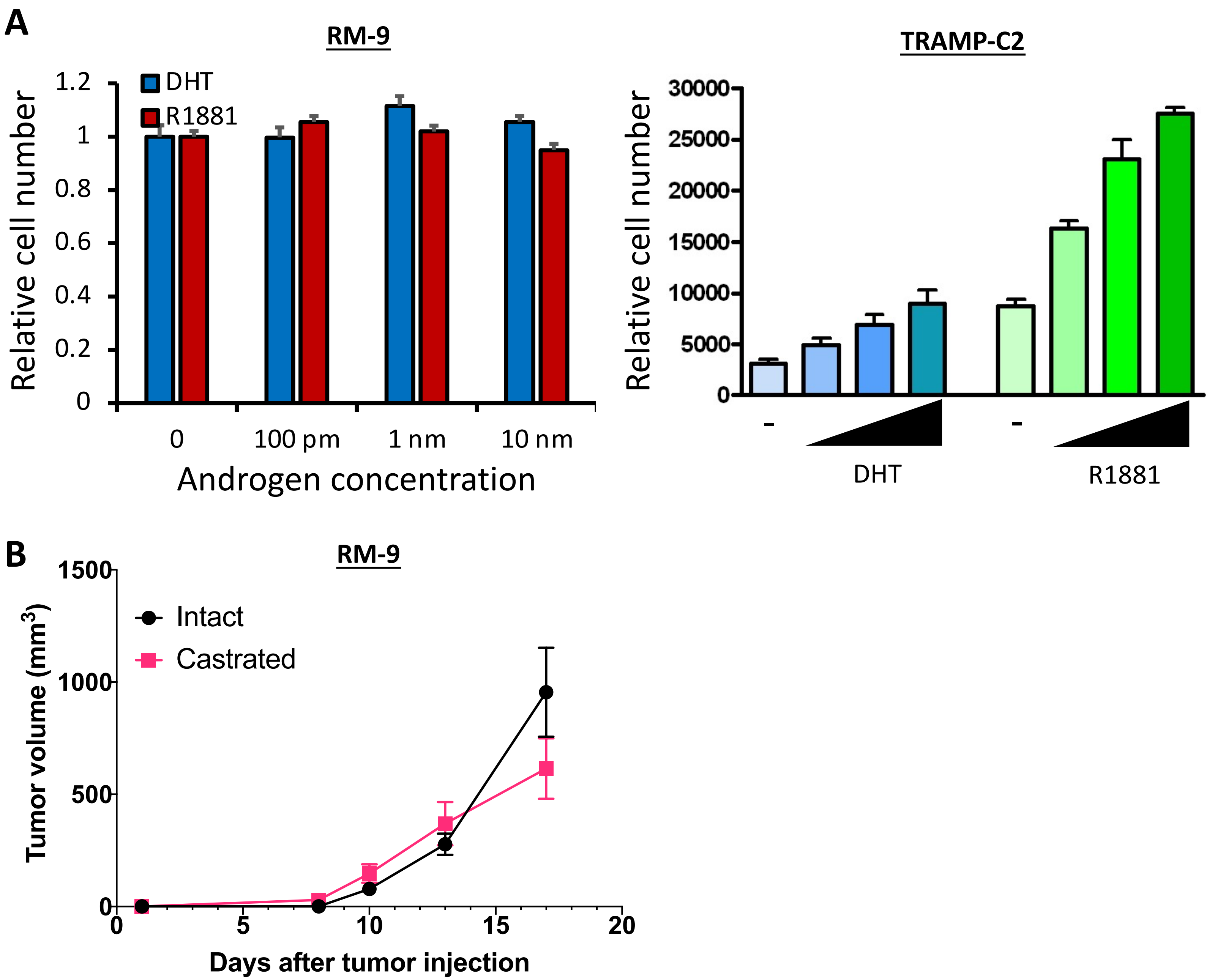

Supplemental Figure S9: Androgen responsive status of TRAMP-C2 and RM-9 cells. (A) RM-9 or TRAMP-C2 murine prostate cancer cells were treated with a dose response (0, 100 pM, 1 nM, 10 nM) of androgens (DHT and R1881) for 7 days and relative cell numbers were quantified using a Hoechst-based DNA dye. Results are expressed as relative cell number + SE. (B) 50,000 RM-9 cells were subcutaneously injected into intact (black; *n*=10) or castrated (red; *n*=7) syngeneic C57BL/6 mice. Tumor growth was monitored by calipers. In our hands, TRAMP-C2 had a low take rate in castrated mice (data not shown).

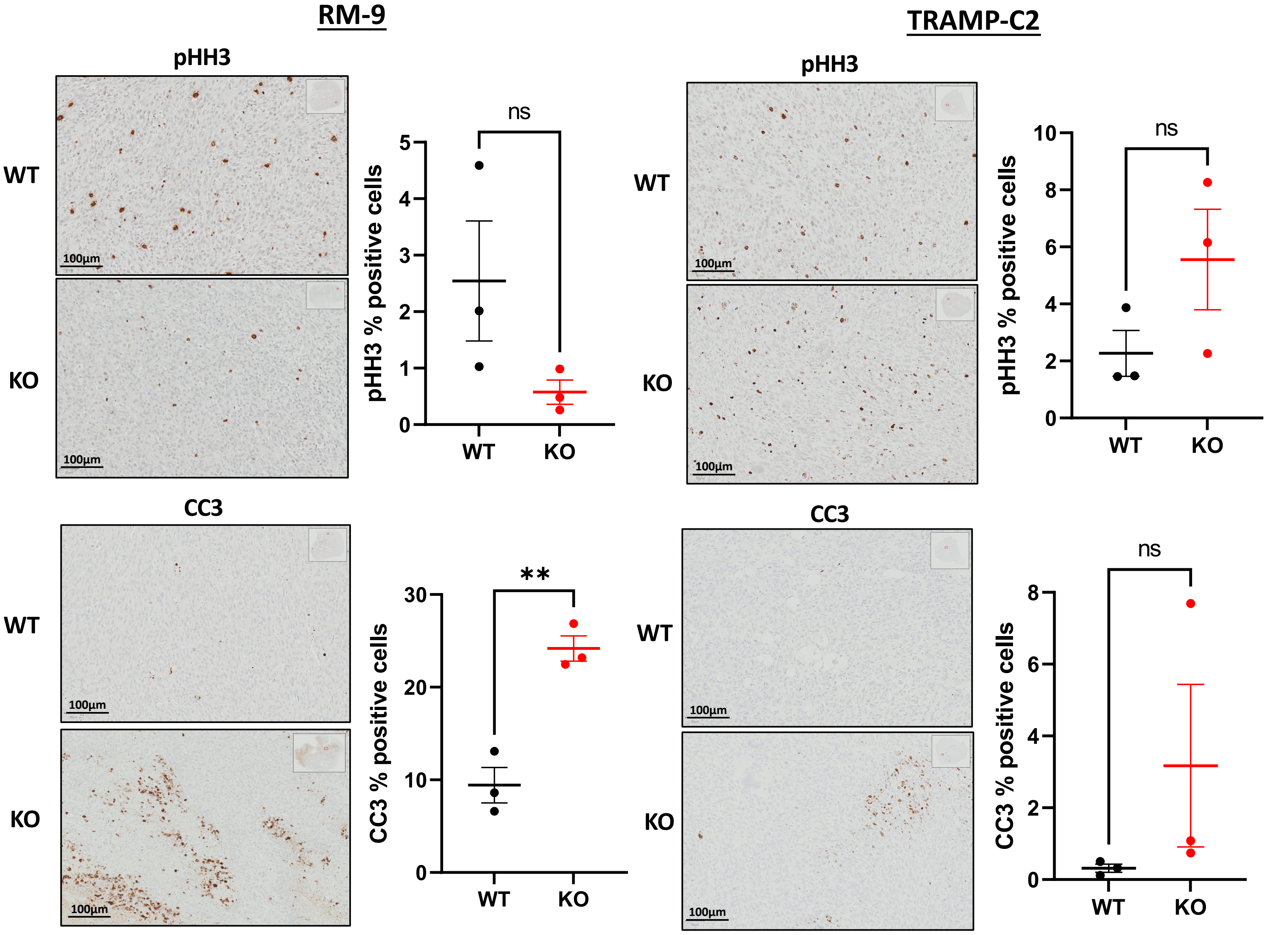

Supplemental Figure S10: Impact of CAMKK2 status on proliferation and apoptosis in syngeneic mouse models of prostate cancer. Phospho-histone H3 (Serine-10) (pHH3) and cleaved caspase-3 (CC3) staining in RM-9 (*left*) and TRAMP-C2 (*right*) syngeneic models. $^{**}P$ value < 0.01; ns = no significance.

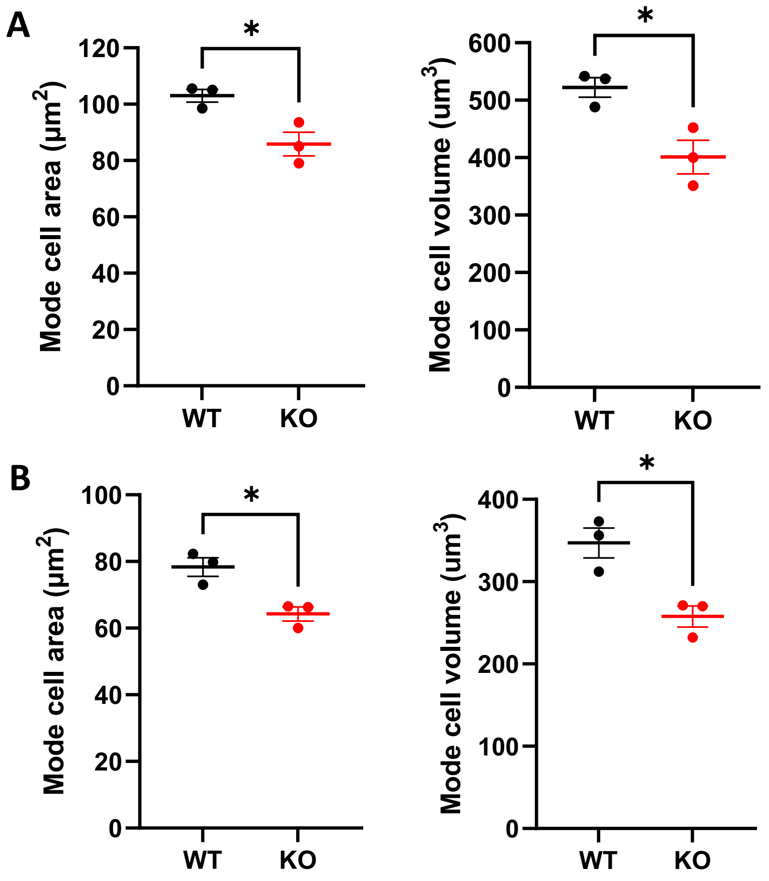

Supplemental Figure S11: Host *Camkk2* ablation decreases cancer cell size in syngeneic mouse models of prostate cancer. (A) RM-9 and (B) TRAMP-C2 mode cell area and mode cell volume were quantified from harvested RM-9 (WT n=3, KO n=3) and TRAMP-C2 (WT n=3, KO n=3) syngeneic tumors. ns=not significant; *P<0.05. For each morphometric analysis, n>1 x 10^6 cells/data point. Average cell areas and volumes are shown in Figure 6.
