# Supplementary material for: Systemic Ablation of Camkk2 Impairs Metastatic Colonization and Improves Insulin Sensitivity in TRAMP Mice: Evidence for Cancer Cell-Extrinsic CAMKK2 Functions in Prostate Cancer
Source: Cells. 2022 Jun 10;11(12):1890. doi: 10.3390/cells11121890 (PMC9221545; doi:10.3390/cells11121890)
Supplement: Supplementary file 1 [file cells-11-01890-s001.zip › Supplementary File S2.pdf]

Gross anatomy images and weights of livers from TRAMP;*Camkk2*+/+ (WT) and TRAMP;*Camkk2*-/- (KO) mice fed normal diet (ND) or high-fat diet (HFD).

Representative images shown in Figure 3.

15-week HFD KO

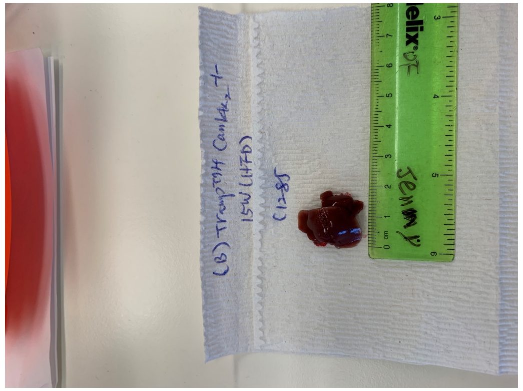

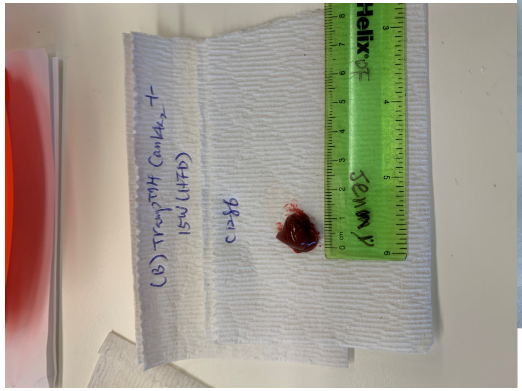

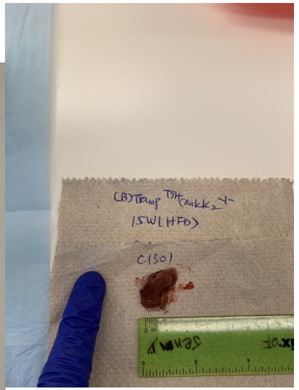

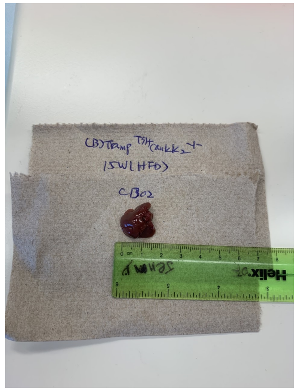

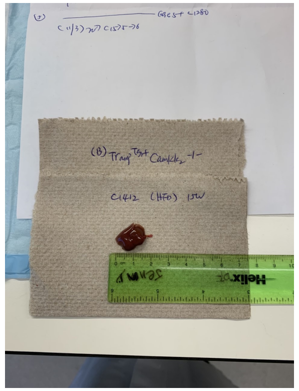

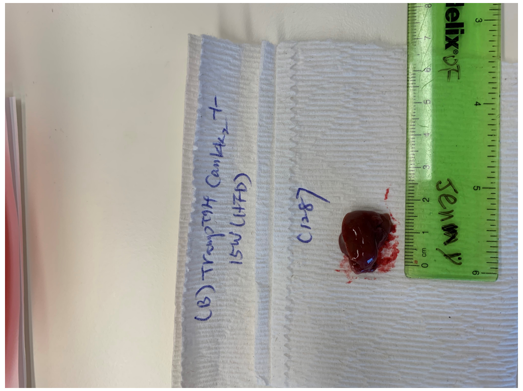

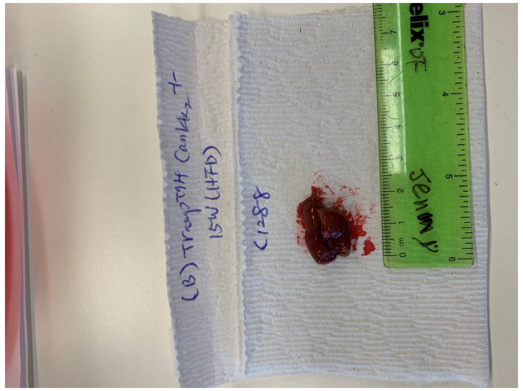

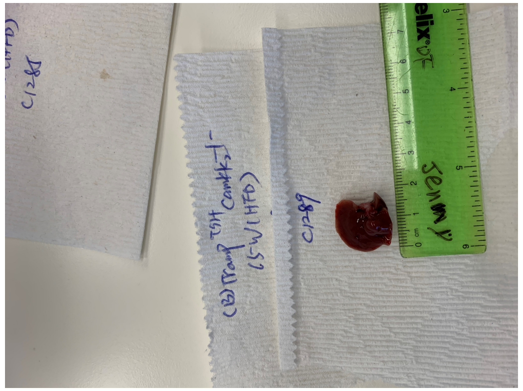

15-week HFD WT

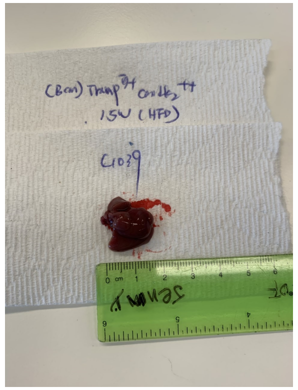
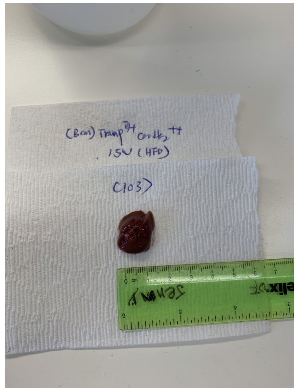
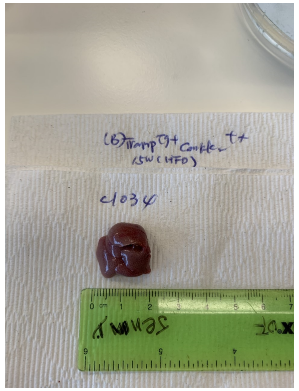
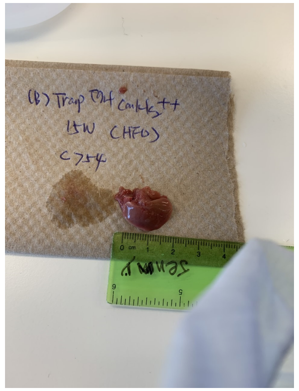
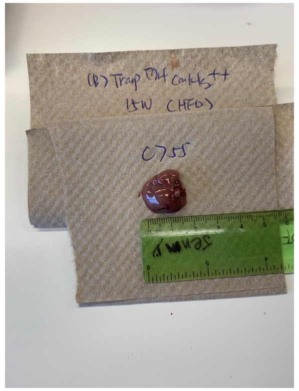
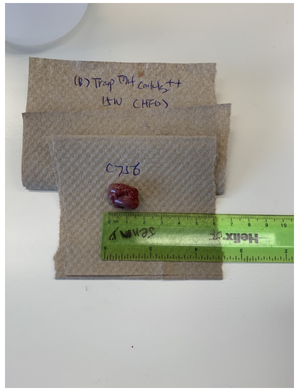
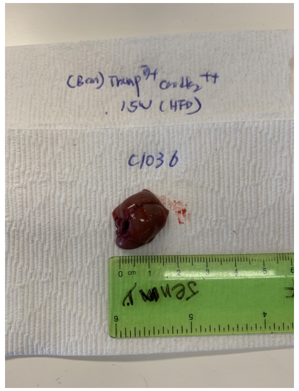
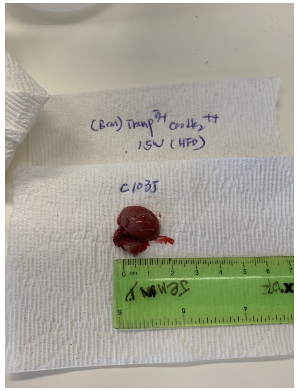
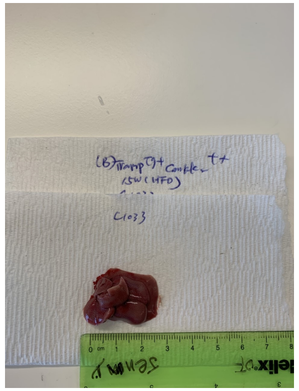

30-week HFD KO

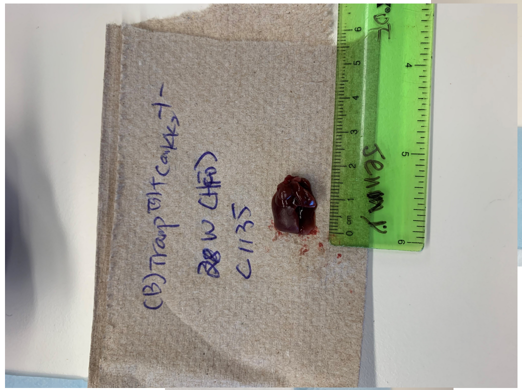

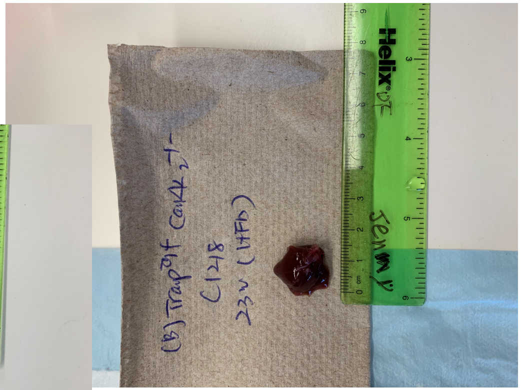

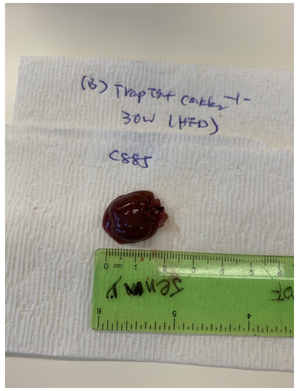

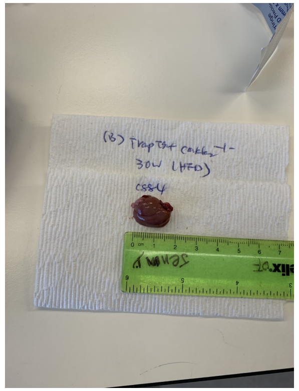

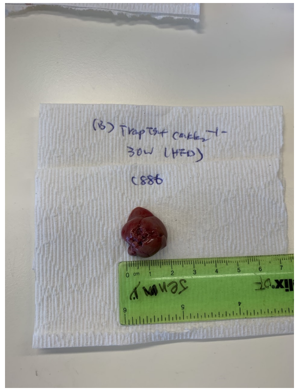

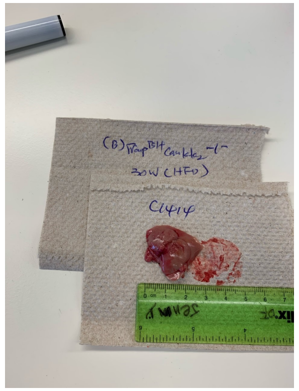

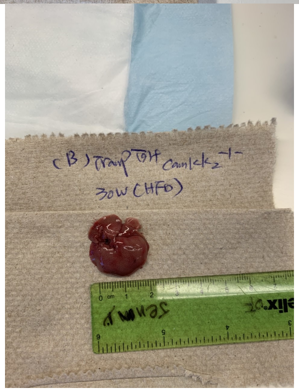

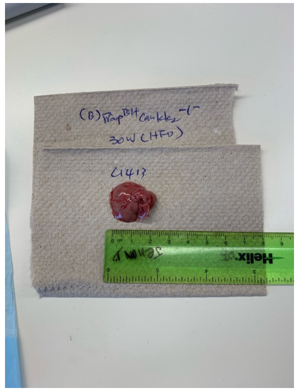

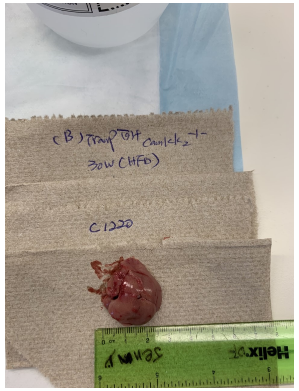

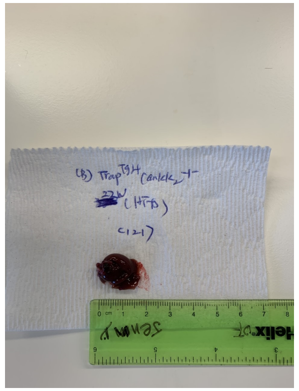

30-week HFD WT

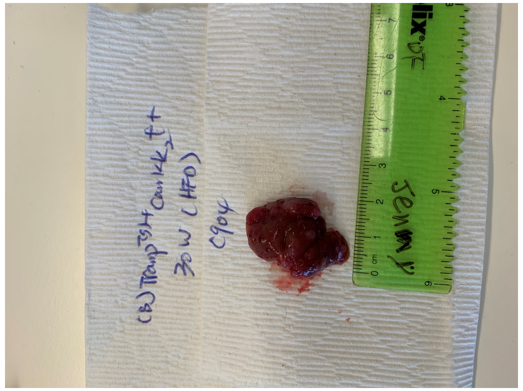

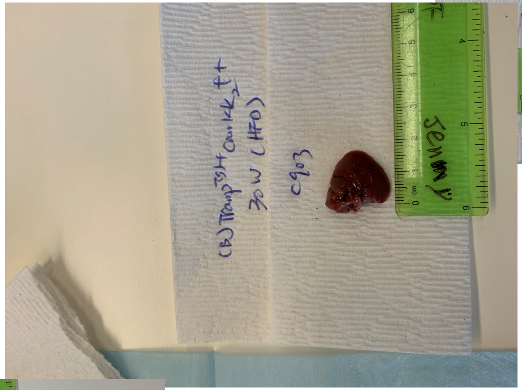

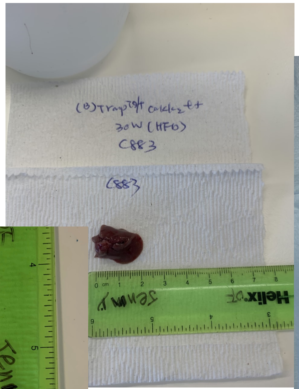

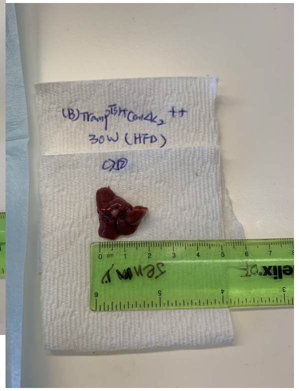

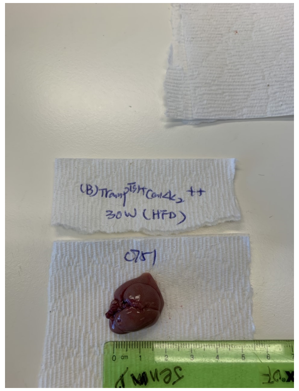

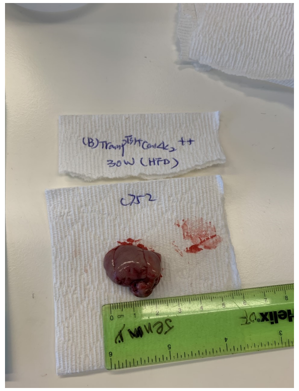

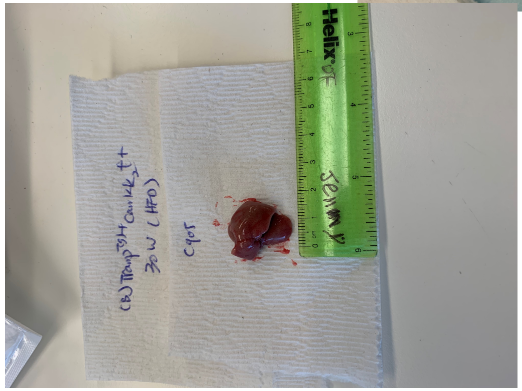

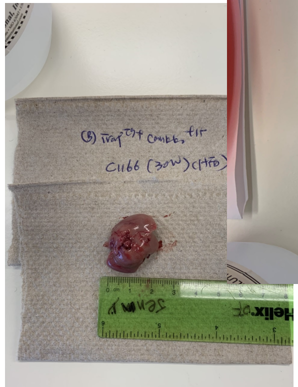

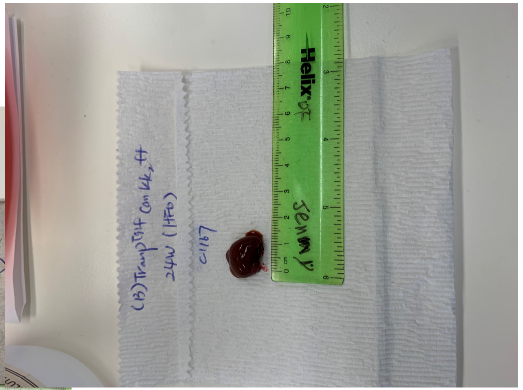

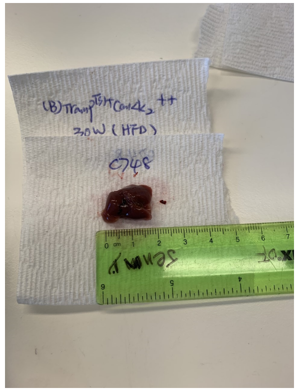

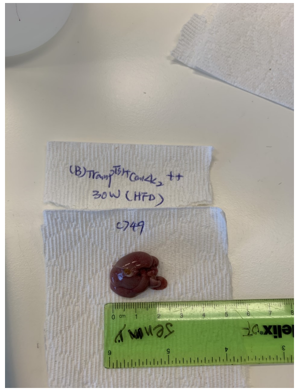

Liver weights

	15 WKS (HFD)										30 WKS (HFD)									
**WT**	1.52	1.54	1.52	2.54	1.85	1.26	1.68	1.44	1.03	1.42	1.07	2.18	1.49	2.3	3.29	2.53	1.42	2.09	3.62	1.16
**KO**	1.51	0.7	1.38	1.43	1.22	1.27	1.2	0.99			1.49	2.54	1.78	0.82	1.02	1.23	2.08	3.93	2.74	2.56

Average 15wks HFD	WT 1.58
	KO 1.2125
